# Supplementary material for: Identification of Novel Candidate CD8+ T Cell Epitopes of the SARS-CoV2 with Homology to Other Seasonal Coronaviruses
Source: Viruses. 2021 May 24;13(6):972. doi: 10.3390/v13060972 (PMC8225204; doi:10.3390/v13060972)
Supplement: Supplementary file 1 [file viruses-13-00972-s001.zip › viruses-1182281-si.pdf]

Supplementary table 1: Homology of different proteins of the SARS-CoV2 with OC43, HKU1, and NL63 corona viruses

Protein	OC43 %	HKU1 %	NL63 %
Spike	29	28	23
Envelope	24	27	20
Membrane	34	34	30
Nucleocapsid	33	30	27
NSP1	19	18	12
NSP2	19	19	18
NSP3	24	23	21
NSP4	41	42	30
NSP5	48	49	44
NSP6	33	32	27
NSP7	46	42	39
NSP8	44	46	47
NSP9	45	44	44
NSP10	52	50	50

NSP11	54	54	34
NSP12	66	67	60
NSP13	68	65	61
NSP14	57	59	53
NSP15	44	46	44
NSP16	67	65	57

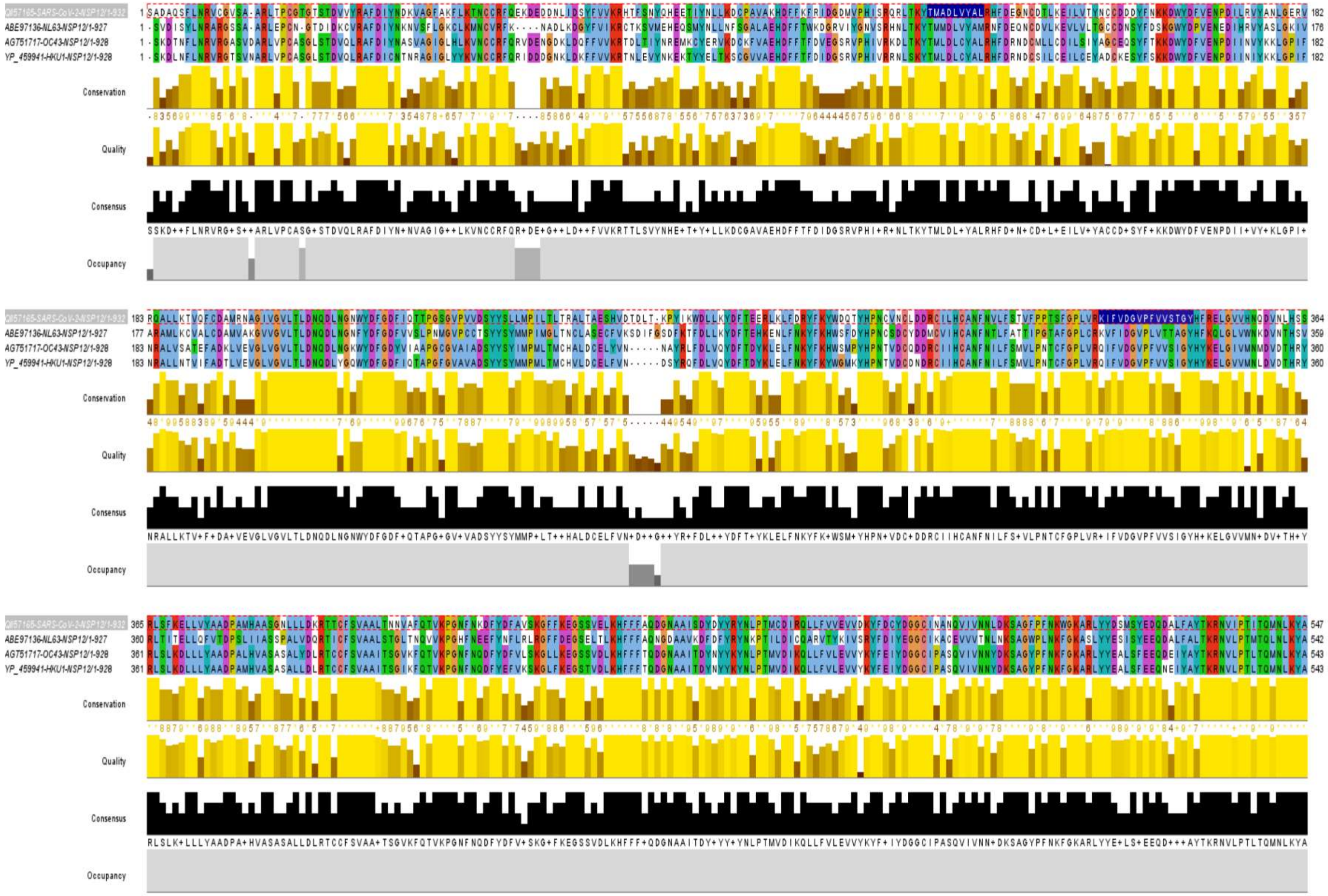
ABE97136-NL63-NSP12/1-927
AG751717-OC43-NSP12/1-928
YP_459941-HKU1-NSP12/1-928
Conservation
Quality
Consensus
Occupancy

NSP12 A

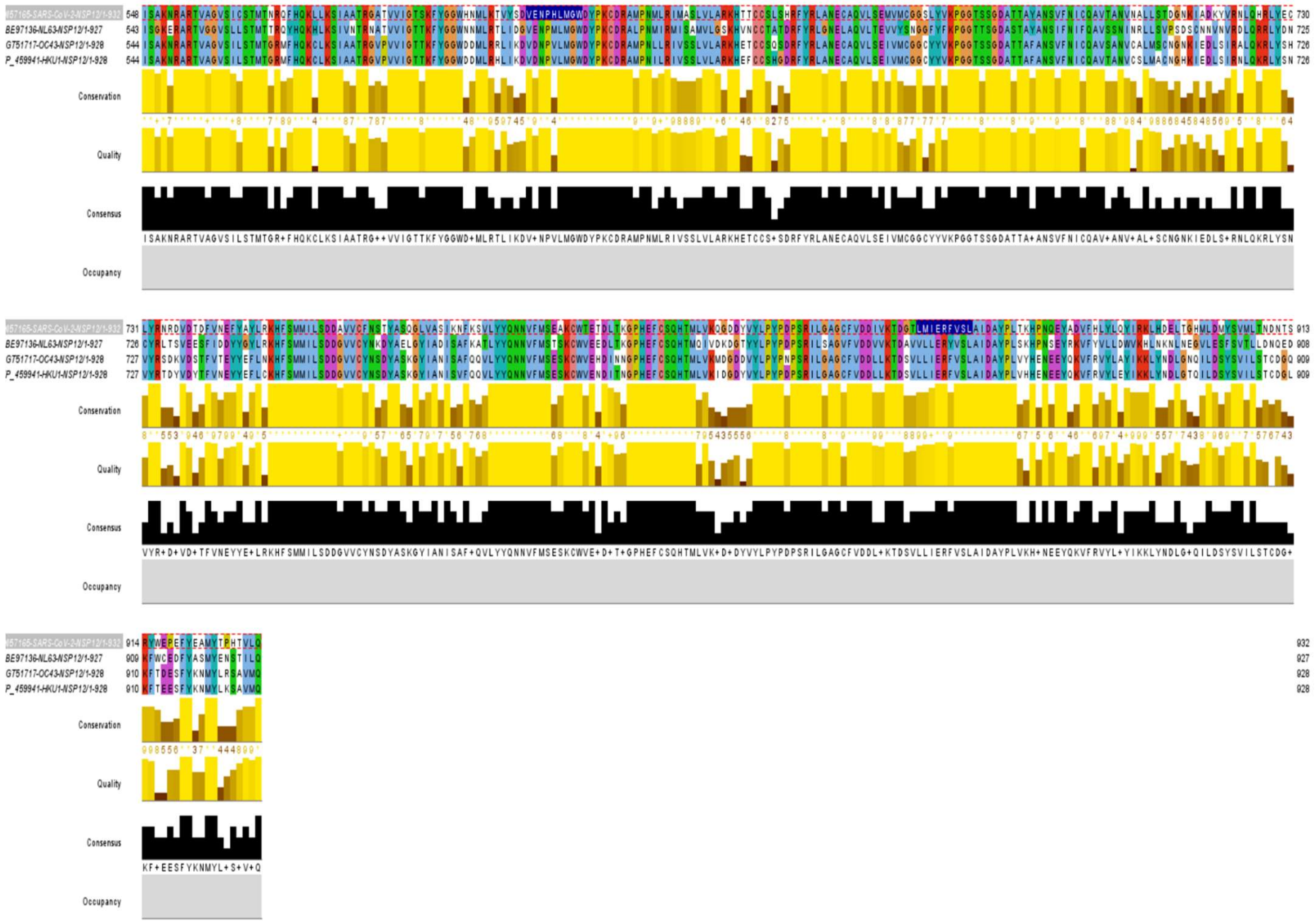

NSP12 B

Supplementary Figure 1: Multiple alignment of 9mer and 10mer peptide sequences identified from SARS-CoV2 NSP12 with OC43, HKU1 and NL63. All peptides shown have a predicted binding score of ≥0.90

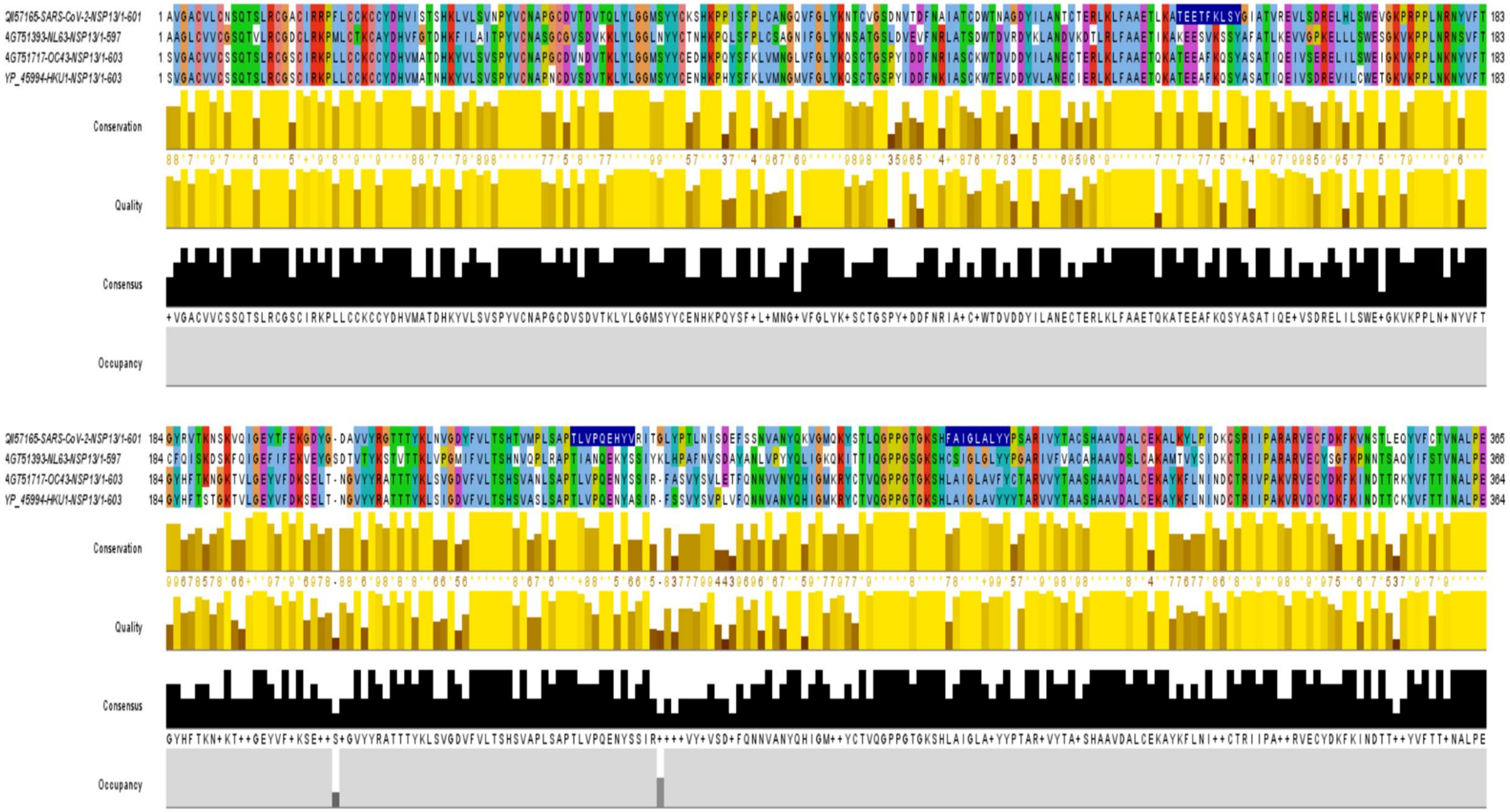
QII57165-SARS-CoV-2-NSP13/1-601
AGT51393-NL63-NSP13/1-597
AGT51717-OC43-NSP13/1-603
YP_45994-HKU1-NSP13/1-603
Conservation
Quality
Consensus
Occupancy

NSP13 A

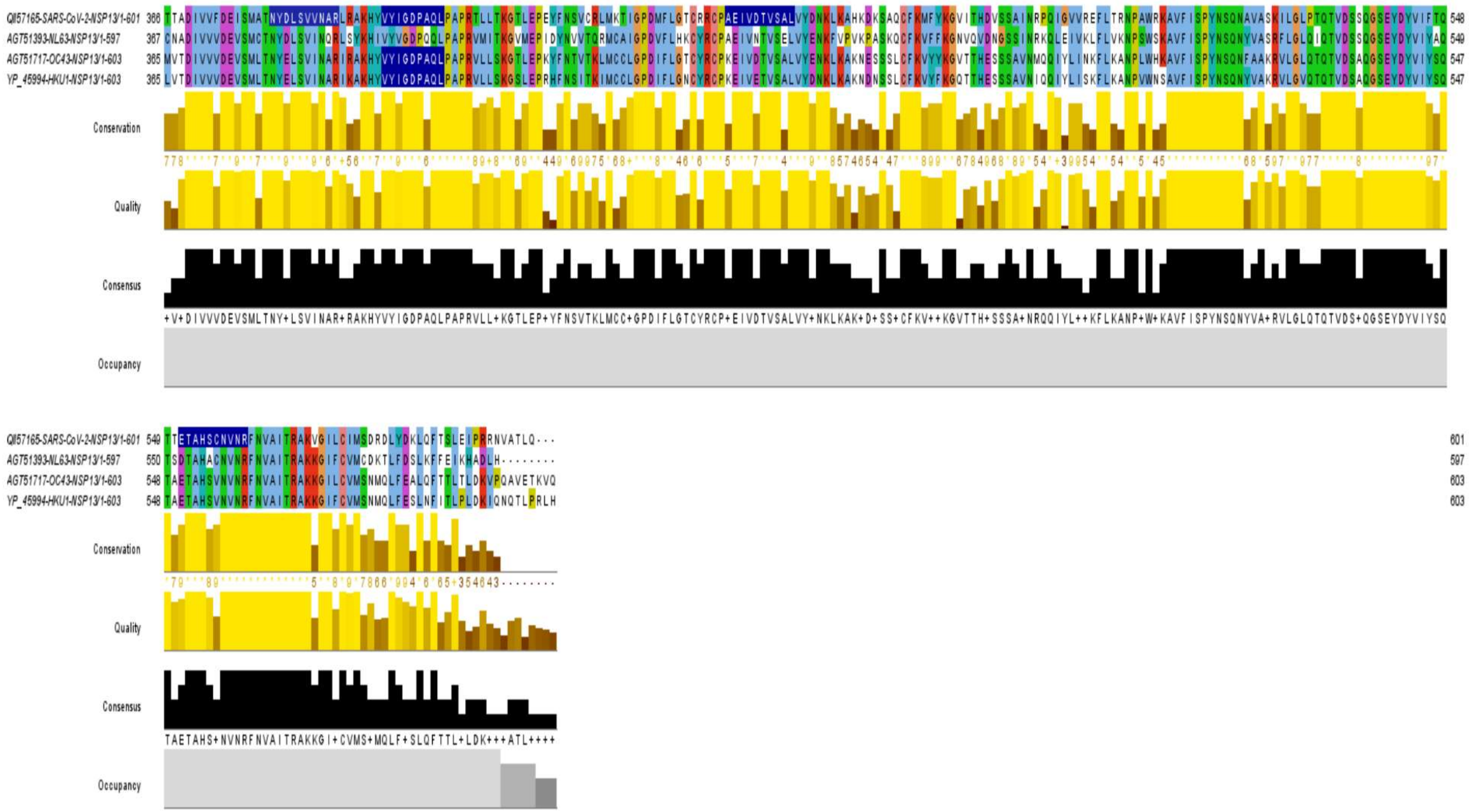

NSP13 B

Supplementary Figure 2: Multiple alignment of 9mer and 10mer peptide sequences identified from SARS-CoV2 NSP12 with OC43, HKU1 and NL63. All peptides shown have a predicted binding score of ≥0.90

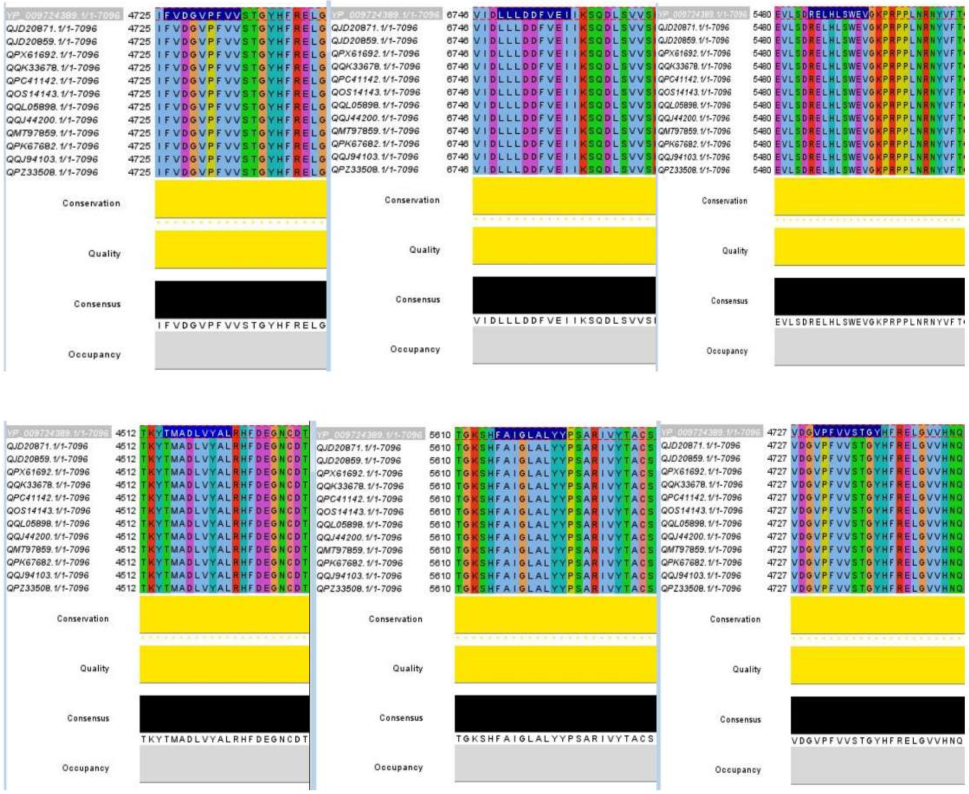

Supplementary figure 3: Conservational analysis of six candidate CD8+ T cell epitopes which had a high degree of homology (>75%) with HCoVs and a binding score of ≥ 0.90 with the SARS-CoV2 variants in different countries, and with the new UK variant (B.1.1.7) and the new South African variant 501.V2. The multiple alignments were performed using Clustal omega, which shows that these epitopes are highly conserved within the different variants of the SARS-CoV2 including the new variants which have emerged in the UK and South Africa.

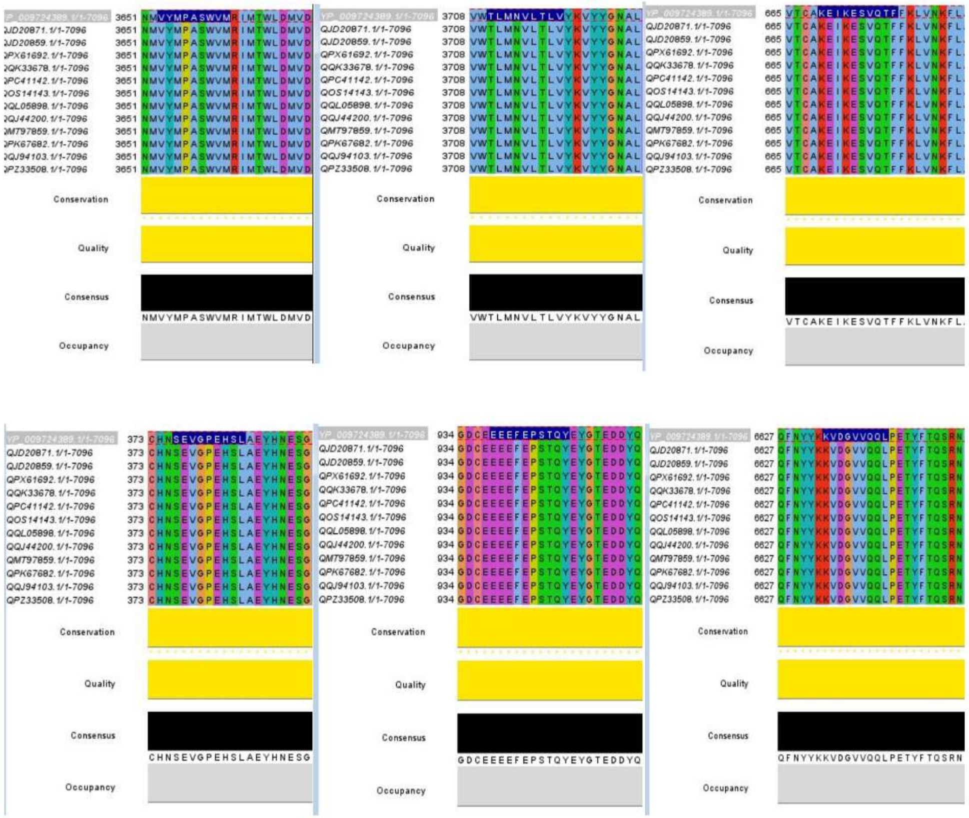

Supplementary figure 4: Conservational analysis of six candidate CD8+ T cell epitopes which were specific to SARS-CoV2 (<25% homology with HCoVs) and a binding score of ≥ 0.90 with the SARS-CoV2 variants in different countries, and with the new UK variant (B.1.1.7) and the new South African variant 501.V2. The multiple alignments were performed using Clustal omega, which shows that these epitopes are highly conserved within the different variants of the SARS-CoV2 including the new variants which have emerged in the UK and South Africa.

Supplementary table 2: Highly conserved candidate CD8+ T cell epitopes which had a high degree of homology (>75%) with HCoVs and a binding score of ≥ 0.90

**Candidate CD8+ T cell 9mer epitopes**									
Protein	HLA Allele	Sequence	Score	OC43	HKU1	NL63	LKR current strain	UK B.1.1.7 variant	South Africa 501.V2 variant
Spike	A*24:02	^{1208}QYIKWPWYI^{1216}	0.95	77	77	77	100	100	100
Membrane	A*24:02	^{95}YFIASFRLF^{103}	0.91	77	77	77	100	100	100
NSP12	A*02:01	^{123}TMADLVYAL^{131}	0.93	77	77	77	100	100	100
NSP12	A*02:06	^{334}FVDGVPFVV^{342}	0.93	100	100	88	100	100	100
NSP12	A*02:01	^{854}LMIERFVSL^{862}	0.91	88	88	55	100	100	100
NSP12	A*02:01	^{334}FVDGVPFVV^{342}	0.90	100	100	88	100	100	100
NSP12	B*44:03	^{608} VENPHLMGW ^{616}	0.99	77	77	88	100	100	100
NSP12	B*35:01	^{337} VPFVVSTGY ^{345}	0.97	88	88	55	100	100	100
NSP13	A*02:01	^{239}TLVPQEHYV^{247}	0.95	77	77	44	100	100	100
NSP13	A*24:02	^{397}VYIGDPAQL^{405}	0.91	100	100	77	100	100	100
NSP13	A*02:06	^{239}TLVPQEHYV^{247}	0.91	77	77	44	100	100	100

NSP13	B*35:01	^{291} FAIGLALYY ^{299}	0.96	77	77	66	100	100	100
NSP13	B*44:03	^{141} TEETFKLSY ^{149}	0.96	77	77	55	100	100	100
NSP13	B*40:02	^{161} RELHLSWEV ^{169}	0.91	77	77	66	100	100	100
NSP14	A*02:01	^{176}NLSDRVVFV^{184}	0.93	77	66	77	100	100	100
NSP14	B*35:01	^{509} WVYKQFDTY ^{517}	0.65	77	77	55	100	100	100
NSP15	A*02:01	^{297}LLLDDFVEI^{305}	0.95	66	88	88	100	100	100
NSP15	B*35:03	^{49} LPVNVAFEL ^{57}	0.96	77	66	88	100	100	100

Supplementary table 3: Highly conserved candidate CD8+ T cell epitopes which were specific to SARS-CoV2 (<25% homology with HCoVs) and a binding score of ≥ 0.90 with the SARS-CoV2 variants in different countries

Candidate 9mer epitopes									
Protein	HLA allele	Sequence	Score	OC43 %	HKU1 %	NL63 %	SRL %	UK %	Africa %
Spike	A*24:02	^{635}VYSTGSNVF^{643}	0.93	22	22	22	100	100	100
Spike	A*02:01	^{109}TLDSKTQSL^{117}	0.91	22	22	33	100	100	100
Spike	B*35:01	^{83}LPFNDGVYF ^{91}	0.98	22	33	11	100	100	100
Spike	B*35:01	^{686}VASQSIIAY ^{694}	0.98	0	0	0	100	100	100
Spike	B*40:01	^{1015}AEIRASANL^{1023}	0.98	22	22	44	100	100	100
Nucleocapsid	B*35:01	^{325}TPSGTWLTY^{333}	0.99	22	22	22	100	100	100
Nucleocapsid	B*44:03	^{322}MEVTPSGTW ^{330}	0.96	11	11	0	100	100	100
NSP1	B*40:02	^{56}VEKGVLPQL^{64}	0.98	22	22	11	100	100	100
NSP1	B*35:01	^{110}HVGEIPVAY^{118}	0.95	22	11	11	100	100	100
NSP2	A*24:02	^{497}TFFKLVNKF^{505}	0.90	33	22	22	100	100	100
NSP2	B*40:01	^{195}SEVGPEHSL^{203}	0.99	11	11	0	100	100	100
NSP2	B*40:01	^{562}GETLPTEVL^{570}	0.99	0	0	0	100	100	100

NSP2	B*44:03	^{52}REHEHEIAW^{60}	0.98	22	22	22	100	100	100
NSP3	A*24:02	^{726}YYTSNPTTF^{734}	0.99	22	22	22	100	100	100
NSP3	A*24:02	^{1349}NYMPYFFTL^{1357}	0.98	33	22	11	100	100	100
NSP3	A*24:02	^{816}YYHTTDPSF^{824}	0.96	11	11	0	100	100	100
NSP3	B*44:03	^{120}EEFEPSTQY^{128}	0.99	11	11	0	100	100	100
NSP3	B*44:03	^{546}QEILGTVSW^{554}	0.99	22	22	0	100	100	100
NSP3	B*40:01	^{1799}AELAKNVSL^{1807}	0.98	0	0	0	100	100	100
NSP4	A*24:02	^{351}FYLTNDVSF^{359}	0.92	22	22	22	100	100	100
NSP4	B*35:01	^{174}NVLEGSVAY^{182}	0.97	11	11	44	100	100	100
NSP6	A*02:01	^{141}TLMNVLTLV^{149}	0.92	0	0	0	100	100	100
NSP6	A*24:02	^{84}VYMPASWVM^{92}	0.91	0	0	11	100	100	100
NSP6	A*24:02	^{115}MYASAVVLL^{123}	0.90	22	22	22	100	100	100
NSP14	A*02:01	^{321}LLADKFPVL^{329}	0.94	11	11	33	100	100	100
NSP15	A*02:06	^{181}KVDGVVQQL^{189}	0.92	22	22	11	100	100	100
Candidate 10mer epitopes									
Protein	HLA Allele	Sequence	Sore	OC43 %	HKU1 %	NL63 %	SRL	UK	Africa
Spike	B*44:02	^{95}TEKSNIIRGW^{104}	0.95	10	0	10	100	100	100

NSP2	B*44:03	^{489}KEIKESVQTF^{498}	0.95	0	0	10	100	100	100
NSP3	B*44:03	^{120}EEEFEPSTQY^{129}	0.98	20	10	0	100	100	100
NSP3	B*35:01	^{502}VPTDNYITTY^{511}	0.94	0	0	0	30	30	30
NSP14	B*35:01	^{42}IPGIPKDMTY^{51}	0.92	20	20	30	100	100	100

Supplementary table 4: The candidate CD8+ T cell epitopes (<25% homology with other HCoVs) and their predicted HLA allele, the published CD8+ T cell epitopes (marked in red) and their HLA restriction.

Candidate epitopes and the published epitopes (marked in red if they overlapped with the candidate epitopes)	Predicted HLA allele	Published alle	Protein
VPTDNYITTY	B*35:01	A*0101	NSP3
YYHTTDPSF	A*24:02	A*0101	NSP3
SALWEIQQVV	A*02:01	A*0201	NSP8
TYVPAQEKNF	A*24:02	A*0201	Spike
GMEVTPSGTW	B*44:03	A*0201	Nucleocapsid
EELKKLLEQW	B*44:03	A*0201	Membrane
VTLAILTALR	A*33:03	A*0201	Envelope
TLDSKTQSL	A*02:01	A*0201	Spike
LPFNDGVYF	B*35:01	A*0301	Spike
NTPKDHIGTR	A*33:03	A*1101	Nucleocapsid
LPFNDGVYF	B*35:01	A*1101	Spike
SRVKNLNSSR	A*33:03	A*2402	Envelope

NYMPYFFTL	A*24:02	A*2402	NSP3
YYTSNPTTF	A*24:02	A*2402	NSP3
GETLPTEVL	B*40:01	B*4001	NSP2
MEVTPSGTWL	B*40:01	B*4001	Nucleocapsid
SELVIGAVIL	B*40:01	B*4001	Membrane
KLNEEIAIIL	A*02:01	B*4403	NSP2
MEVTPSGTW	B*44:03	B*4403	Nucleocapsid
QEILGTVSW	B*44:03	B*4403	NSP3

Supplementary table 5: The candidate CD8+ T cell epitopes (?75% homology with other HCoVs) and their predicted HLA allele, the published CD8+ T cell epitopes (marked in red) and their HLA restriction.

Candidate epitopes and the published epitopes (marked in red if they overlapped with the candidate epitopes)	Predicted HLA allele	Published alle	Protein
VLNDILSRL	A*02:01	A*0201	Spike
NYDLSVVNAR	A*33:03	A*1101	NSP13
WVYKQFDTY	B*35:01	A*1101	NSP14

QYIKWPWYI	A*24:02	A*2402	Spike
VYIGDPAQL	A*24:02	A*2402	NSP13
NPKTPKYKF	B*35:01	B*0801	NSP5
EEAIRHVRAW	B*44:02	B*4403	NSP14
